# The Two-Component Signal Transduction System ArlRS Regulates *Staphylococcus epidermidis* Biofilm Formation in an *ica*-Dependent Manner

**DOI:** 10.1371/journal.pone.0040041

**Published:** 2012-07-27

**Authors:** Yang Wu, Jiaxue Wang, Tao Xu, Jingran Liu, Wenqi Yu, Qiang Lou, Tao Zhu, Nianan He, Haijing Ben, Jian Hu, Friedrich Götz, Di Qu

**Affiliations:** 1 Key Laboratory of Medical Molecular Virology of the Ministry of Education and Ministry of Public Health, Institute of Medical Microbiology and Institute of Biomedical Sciences, Shanghai Medical College of Fudan University, Shanghai, P.R. China; 2 Microbial Genetics, University of Tübingen, Tübingen, Germany; 3 Department of Ultrasound, Zhongshan Hospital of Fudan University, Shanghai, China; National Institutes of Health, United States of America

## Abstract

Due to its ability to form biofilms on medical devices, *Staphylococcus epidermidis* has emerged as a major pathogen of nosocomial infections. In this study, we investigated the role of the two-component signal transduction system ArlRS in regulating *S. epidermidis* biofilm formation. An ArlRS-deficient mutant, WW06, was constructed using *S. epidermidis* strain 1457 as a parental strain. Although the growth curve of WW06 was similar to that of SE1457, the mutant strain was unable to form biofilms *in vitro*. In a rabbit subcutaneous infection model, sterile disks made of polymeric materials were implanted subcutaneously followed with inoculation of WW06 or SE1457. The viable bacteria cells of WW06 recovered from biofilms on the embedded disks were much lower than that of SE1457. Complementation of arlRS genes expression from plasmid in WW06 restored biofilm-forming phenotype both *in vivo* and *in vitro*. WW06 maintained the ability to undergo initial attachment. Transcription levels of several genes involved in biofilm formation, including *icaADBC*, *sigB*, and *sarA*, were decreased in WW06, compared to SE1457; and *icaR* expression was increased in WW06, detected by real-time reverse-transcription PCR. The biofilm-forming phenotype was restored by overexpressing *icaADBC* in WW06 but not by overexpressing *sigB*, indicating that ArlRS regulates biofilm formation through the regulation of *icaADBC*. Gel shift assay showed that ArlR can bind to the promoter region of the *ica* operon. In conclusion, ArlRS regulates *S. epidermidis* biofilm formation in an *ica*-dependent manner, distinct from its role in *S. aureus*.

## Introduction

*Staphylococcus epidermidis* is an opportunistic pathogen that normally colonizes human skin and mucosal surfaces. Over the past two decades, *S. epidermidis* has emerged as a major pathogen of nosocomial infections, particularly infections involving indwelling medical device [1]–[3]. *S. epidermidis* pathogenesis is associated with its ability to colonize polymer surfaces to form multilayered biofilms, which impair the efficacy of antibiotic treatments and serve to protect the bacteria from the host immune system [4], [5].

Staphylococcal biofilm formation is a complicated process that is regulated by multiple regulatory factors including SigB, *agr*, SarA, and two-component signal transduction systems (TCSs) [6]–[10]. TCSs mediate a diverse range of adaptive responses to environmental stresses and play a vital role in bacterial pathogenesis [11]–[14]. In *S. epidermidis*, whereas the TCSs Agr, LytSR and SaeRS are known to be involved in biofilm formation [15]–[17], the role of the ArlRS TCS remains unclear.

**Table 1:** Plasmids and bacterial strains.

	Discription
**Plasmids**	
pBT2	temperature-sensitive *E.coli-Staphylococcus* shuttle vector
pTX*icaADBC*	*icaADBC* cloned in pTX15, a xylose inducible plasmid
pTX*sigB*	*sigB* cloned in pTX15
pCN*arlS*	*arlS* cloned in pCN51, a Cd^2+^ inducible plasmid
pCN*arlRS*	*arlRS* cloned in pCN51
**Bacterial strains**	
RP62A	a standard strain of *S. epidermidis*, biofilm positive
ATCC12228	a standard strain of *S. epidermidis*, biofilm negative
SE1457	a clinical strain of *S. epidermidis*, biofilm positive
WW06	a *arlS* gene deletion mutant of SE1457, in which ArlR cannot be translated
ParlS	WW06 complemented with the plasmid pCN*arlS*
ParlRS	WW06 complemented with the plasmid pCN*arlRS*
PsigB	WW06 complemented with the plasmid pTX*sigB*
PicaADBC	WW06 complemented with the plasmid pTX*icaADBC*
Δ*icaC*	*icaC* gene deletion mutant of SE1457
Δ*atlE*	*atlE* gene deletion mutant of SE1457
icaCpRS	Δ*icaC* complemented with the plasmid pCN*arlRS*

The ArlRS TCS was first identified in *Staphylococcus aureus* [18], [19]. ArlRS strikingly modifies the extracellular proteolytic activity of *S. aureu*s and is a regulator of virulence gene expression. Mutations in either *arlR* or *arlS* increase the production of Protein A, α-toxin, β-hemolysin, lipase, coagulase and serine protease. It was suggested that ArlRS may interact with both *agr* and *sarA* regulatory loci to modulate the virulence regulation network. A recent study found ArlRS positively regulate *S. aureus* capsule formation in a sigma B dependent manner [20]. Transcriptional profile analysis showed that in *S. aureus*, ArlRS positively regulated virulence factor genes such as *sdrC, sdrD* and *sdrE*, and functioned as a repressor of several toxin genes including *lukD, lukE, phlC* and *hlgC* [21]. Furthermore, ArlRS was found to be involved in *S. aureus* biofilm formation. An *arlS* gene transposition mutant of *S. aureus* clinical strain MT23142 exhibited an increased ability to form biofilms on polymer surfaces, resulting from altered bacterial autolysis and peptidoglycan hydrolase activity [18]. Deletion of the *arlRS* locus in *S. aureus* clinical strain 15981 enhanced initial cell attachment and biofilm formation when cultured in Hussain-Hastings-White-modified medium (HHWm) [22].

Staphylococcal biofilm formation occurs in two steps: bacterial cells attach to the material surface; intercellular adhesion between bacterial cells forms multi-layered structures. In the second step, poly-N-acetylglucosamine (PNAG) in *S. aureus* or polysaccharide intercellular adhesin (PIA) in *S. epidermidis* synthesized by *ica* operon-encoded enzymes play a key role [5], [23].

**Table 2:** Primers used in this study.

Name	Primers	Applications	
ICAP-S	5′-CTTTCAATTCTAAAATCTCCCCCTT-3′	*icaADBC* promoter	
ICAP-AS	5′-TTTTTCACCTACCTTTCGTTAGTTA-3′	*icaADBC* promoter	EMSA
ICAA-S	5′-GAGGGAATCAAACAAGCA-3′	*icaA* fragment	
ICAA-AS	5′-AGGCACTAACATCCAGCA-3′	*icaA* fragment	
U-S	5′-CCGGAATTCTGGAACCGTATCGACAC-3′	*arlS* (upstream)	
U-A	5′-CGCGGATCCGGTGGTATGATTCAGGTTG-3′	*arlS* (upstream)	Gene knockout
D-S	5′-AACTGCAGCGAATCACATACCCTACG-3′	*arlS* (downstream)	
D-A	5′-CTAGCTAGCCTTGTATGTGGGGGGAAT-3′	*arlS* (downstream)	
U-S’	5′-CGCTAAAGAAATACGTTGT-3′	*arlS* (upstream)	
U-A’	5′-GCGCTAGGGACCTCTTTA-3′	*arlS* (upstream)	
D-S’	5′-CTATTGTGAGTTATTAGTGG-3′	*arlS* (downstream)	
D-A’	5′-AAGTGTCAAAGGTCTACTG-3′	*arlS* (downstream)	Mutant validation
erm-S	5′-CTATTGTGAGTTATTAGTGG-3′	*ermB* cassette	
erm-A	5′-GCGCTAGGGACCTCTTTA-3′	*ermB* cassette	
arlS-S'	5′-TCAACCTGAATCATACCACC-3′	*arlS* amplification	
arlS-A'	5′-GATGCTTATTACGACGCTCAT-3′	*arlS* amplification	
sarA-S	5′-TTCAAAAATCAATGACTGCT-3′	*sarA* amplification	
sarA-AS	5′-TTCCTCTTCTTTATTCTCAC-3′	*sarA* amplification	
sigB-S	5′-TCACCTGAACAAATTAACCAATG-3′	*sigB* amplification	
sigB-AS	5′-CACCTATTAGACCAACCATACC-3′	*sigB* amplification	
arlS-S	5′-ATTATTCAAGGTCATCTCAA-3′	*arlS* amplification	
arlS-A	5′-ATCTCGCTATTTATGTCTAC-3′	*arlS* amplification	
icaA-S	5′-TTATTGGTTGTATCAAGCGAAGTC-3′	*icaA* amplification	Real-time PCR
icaA-AS	5′-TCCTCAGTAATCATGTCAGTATCC-3′	*icaA* amplification	
luxS-S	5′-GAGCAGATGATTATATTGT-3′	*luxS* amplification	
luxS-AS	5′-TGTTAGATTCACTTGTTG-3′	*luxS* amplification	
rsbU-S	5′-TCTCTTCATACAGTCCAT-3′	*rsbU* amplification	
rsbU-AS	5′-ATAGGTTCAGGTATTCCA-3′	*rsbU* amplification	
icaR-S	5′-GGAGCACTAGATAATTGAACAT-3′	*icaR* amplification	
icaR-S	5′-CATTGACGGACTTTACCAG-3′	*icaR* amplification	
gyrB-S	5′-TAGTATTGACGAGGCATTAGCA-3′	*gyrB* amplification	
gyrB-AS	5′-TATCCGCCACCTCCGA-3′	*gyrB* amplification	

Toledo-Arana’s study demonstrated that although the accumulation of PNAG was increased in the *arlRS* mutant of *S. aureus*, deletion of *icaADBC* did not result in biofilm abolishment. This finding indicates ArlRS may be involved in *S. aureus* biofilm formation in an *ica*-independent manner [22].

Here, we demonstrate for the first time that the ArlRS plays an important role in the regulation of *S. epidermidis* biofilm formation, and acts in an *ica*-dependent manner distinct from the role of ArlRS in *S. aureus* biofilm formation.

**Figure 1:**
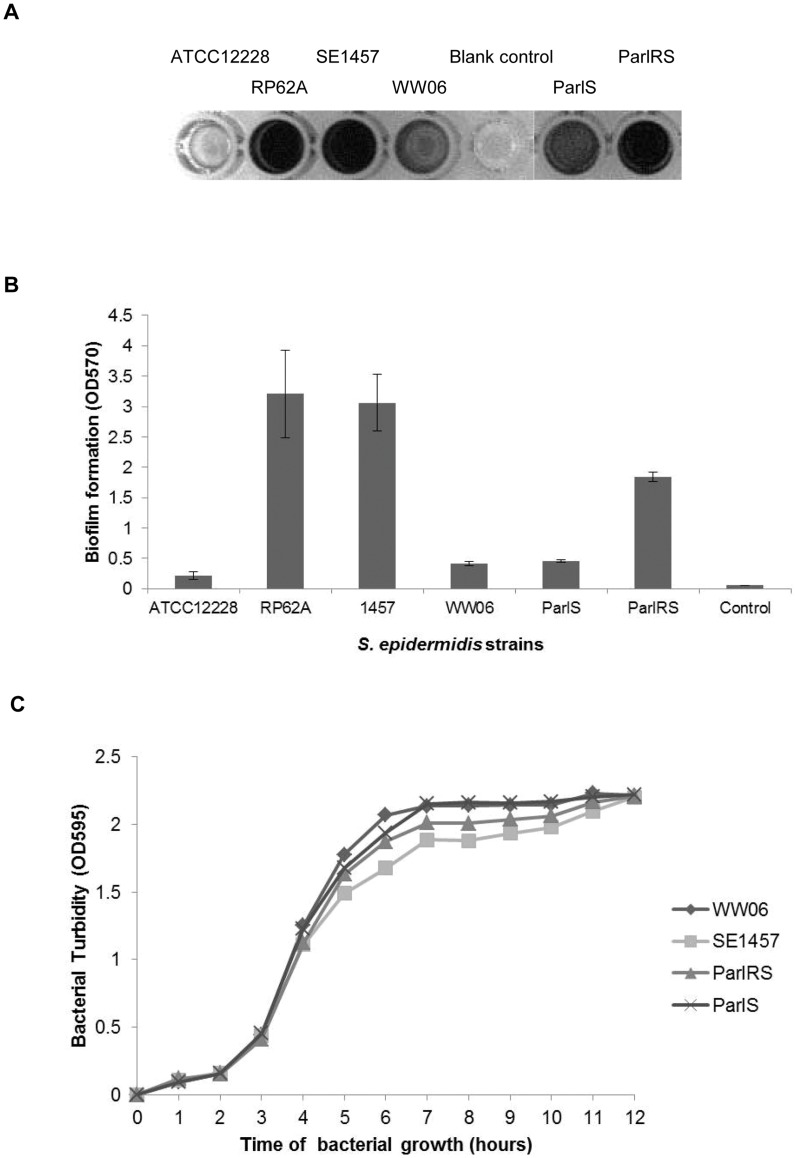
Effect of ArlRS deficiency on *S.epidermidis* growth and biofilm formation *in vitro*. (A)Semi-quantitative detection of biofilms formed by SE1457, WW06, ParlS and ParlRS over a 24 h period in 96-well polystyrene microtiter plates: The biofilms were stained with 2% crystal violet. (B) The crystal violet was dissolved in ethanol and the absorbance was determined at 570 nm. The mean value ± standard deviation for each strain is shown. (C) Growth curves of *S. epidermidis* strains: Overnight cultures of SE1457, WW06, ParlS and ParlRS were diluted to OD_595_ = 0.08 in 150 ml fresh TSB and incubated at 37°C with shaking.The value of OD_595_ was monitored hourly. The curves represent the means of three independent experiments with standard deviation (SD). *Staphylococcus epidermidis* strain ATCC12228, a non-biofilm forming control; RP62A, a biofilm positive control.

## Materials and Methods

### Ethics Statement

All procedures performed on rabbits were conducted according to relevant national and international guidelines (the Regulations for the Administration of Affairs Concerning Experimental Animals, China, and the NIH Guide for the Care and Use of Laboratory Animals) and were approved by the Institutional Animal Care and Use Committee (IACUC) of Shanghai Medical College of Fudan University (IACUC Animal Project Number: 20110307-066).

**Figure 2:**
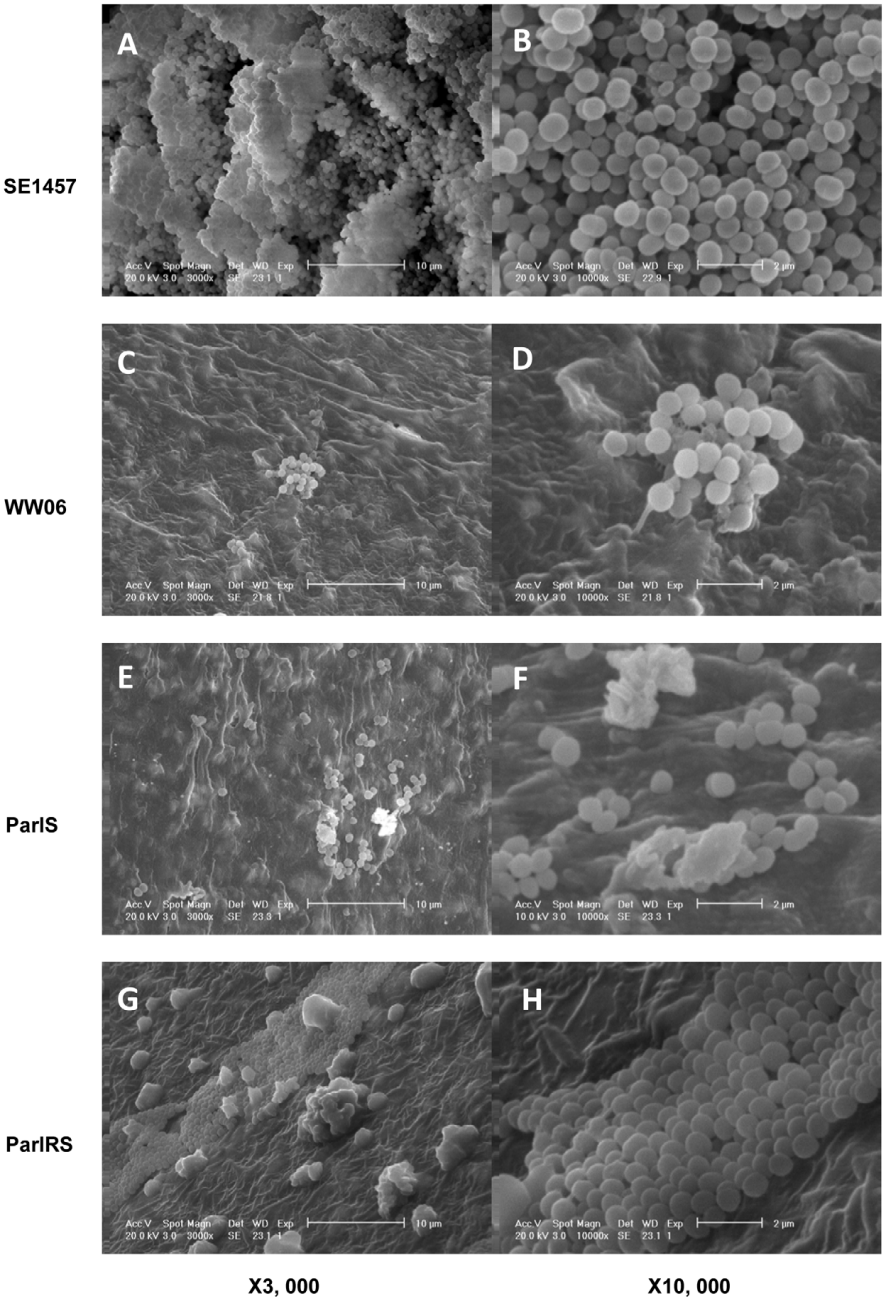
Biofilms formed on catheter fragments surfaces observed by scanning electron microscope (SEM). *S. epidermidis* strains WW06, ParlS, ParlRS and SE1457 were incubated with central vein catheter fragments in 96-well plates. After incubation at 37°C for 24 h, biofilm formed on the surface of the catheter fragments were observed under a transmission electron microscope (XL-30, Philips). Images were obtained at different magnifications (x3000, x10000) for biofilms formed by SE1457 (a, b), WW06 (c, d), ParlS (e, f) and ParlRS(g, h).

### Bacterial Strains, Plasmids and Growth Media

Bacterial strains and plasmids used in this study are listed in Table 1. B-Medium and Tryptic soy broth (TSB, Oxoid, Cambridge, UK) were used for *S. epidermidis* cultivation and biofilm formation. Media were supplemented with erythromycin (10 µg/ml), ampicillin (100 µg/ml), tetracycline (10 µg/ml) or chloramphenicol (10 µg/ml) when appropriate for purposes of selection.

**Figure 3:**
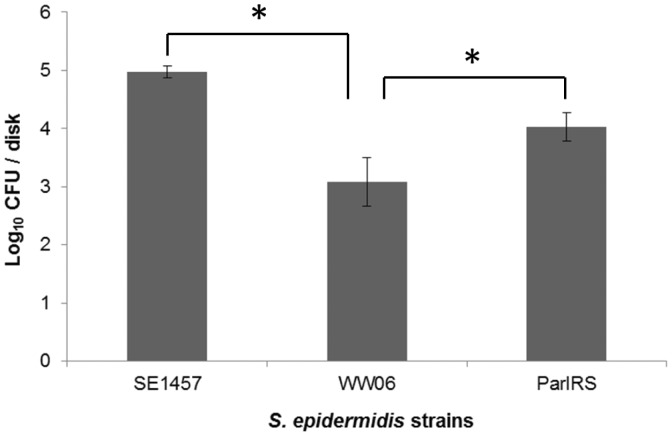
Influence of ArlRS deficiency on viable *S. epidermidis* cells recovery from the implanted disks in the rabbit model. Biofilms of SE1457, WW06 and ParlRS were formed *in vivo* on sterile polyethylene disks that were implanted subcutaneously in three New Zealand White female rabbits. Three days after infection, the implants were removed. Biofilms were scraped from the disks and CFUs of the viable bacteria recovered from the biofilms were determined and expressed as mean ± standard deviation. Asterisks denote statistically significant difference, P<0.001.

### Construction of *arlS* Gene Knockout Mutant and Complementation Strains

The *arlS* gene in *S. epidermidis* SE1457 was deleted using the temperature-sensitive vector pBT2 [24]. Briefly, an erythromycin-resistance cassette (*ermB*) was inserted into the pBT2 plasmid. Then the regions flanking *arlS* gene were amplified by PCR and inserted into pBT2-*ermB*. Primers for PCR were designed according to the genomic sequence of *S. epidermidis* RP62A (GenBank accession number CP000029). Primer sequences are listed in Table 2. The recombinant plasmid, designated pBT2-*arlS*, was transformed by electroporation into *S. aureus* strain RN4220 then into SE1457. A procedure for allelic displacement of the *arlS* gene was performed as previous described [24], [25]. The mutant, designated WW06, was verified by PCR, RT-PCR and direct sequencing. Complementation studies were performed using a vector pCN51 [26] with a shine-dalgarno sequence plus either the *arlS* gene alone or *arlRS* genes. The resulting plasmids, pCN-*arlS* and pCN-*arlRS*, were transformed by electroporation into WW06, forming two complementary strains, ParlS and ParlRS, respectively.

**Figure 4:**
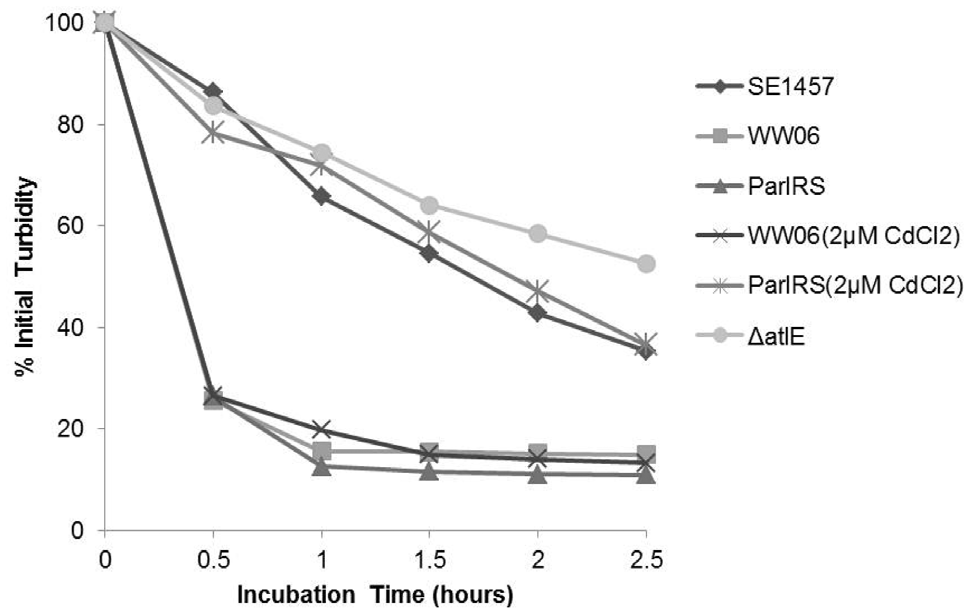
Effect of *arlRS* mutation on *S. epidermidis* Triton X-100-induced autolysis. SE1457, WW06, ParlRS and Δ*atlE* were cultured in TSB containing 1 M NaCl at 37°C to exponential phase (OD_580_ = 0.7). WW06 and ParlRS were also cultured in TSB containing 1 M NaCl and 2 µM CdCl_2_. Triton X-100 induced autolysis of the strains was determined by measuring the change in the value of OD_595_ at 30 min intervals; results are expressed as lysis percentages. Percent lysis was calculated as follows: [(OD_0_-ODat time *t*/OD_0_)] X 100%]. Experiments were carried out three times independently.

### Expression of Recombinant ArlR (rArlR) and Preparation of the anti-rArlR Antiserum

The *arlR* gene was amplified from the genomic DNA of SE1457 and inserted into the vector pET-28a(+) to obtain the recombinant plasmid pET-*arlR*. The recombinant plasmid was then transformed into *Escherichia coli* BL21 (DE3). The expressed His-tagged ArlR protein was purified using the ProBond™ Purification System (Invitrogen, Carlsbad, USA) according to the manufacturer’s instructions.

Five Balb/c mice were immunized with 5 μg purified rArlR coupled with aluminum hydroxide adjuvant by intraperitoneal injection, and boosted after four weeks. The antiserum was collected and anti-rArlR antibodies were detected with an immune dot-blot.

**Figure 5:**
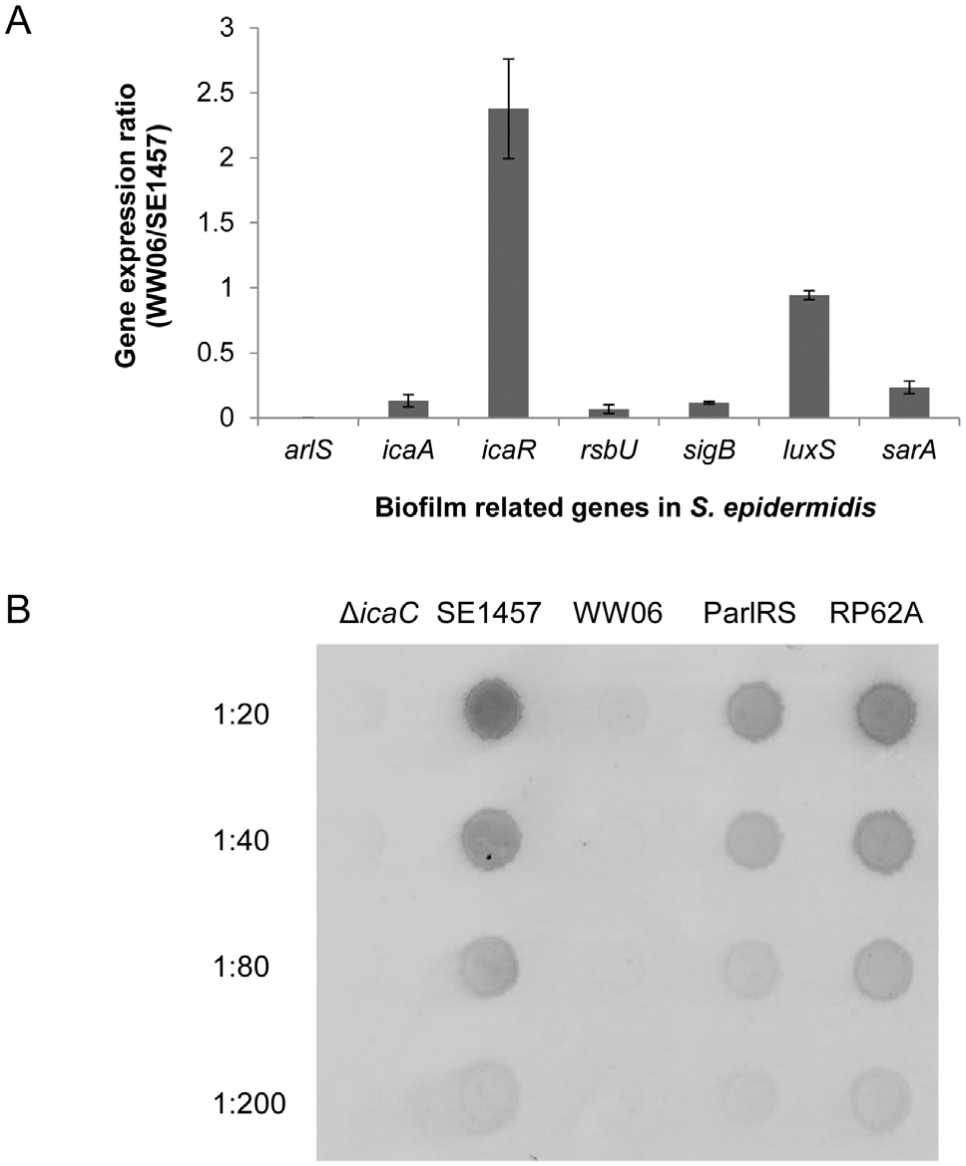
Regulation of expression of biofilm-related genes by ArlRS. (A) Effect of ArlRS deficiency on transcription levels of the biofilm-related genes. Gene expression profiles of the *arlRS*-deficient strain WW06 and the wild-type parent strain SE1457 in the mid-log growth phase were analyzed using RT-qPCR. The experiment was performed in triplicate and the expression ratios of the biofilm-related genes in WW06 and SE1457 are represented as mean ± standard deviation. (B) Effect of *arlRS* mutation on PIA synthesis. PIA was extracted from cells grown for 24 h under biofilm conditions in 6-well cell culture plates. PIA was detected by dot blot analysis using wheat germ agglutinin coupled to horseradish peroxidase. The PIA-deficient *S. epidermidis* mutant Δ*icaC* was used as a negative control, and RP62A was used as a positive control.

**Figure 6:**
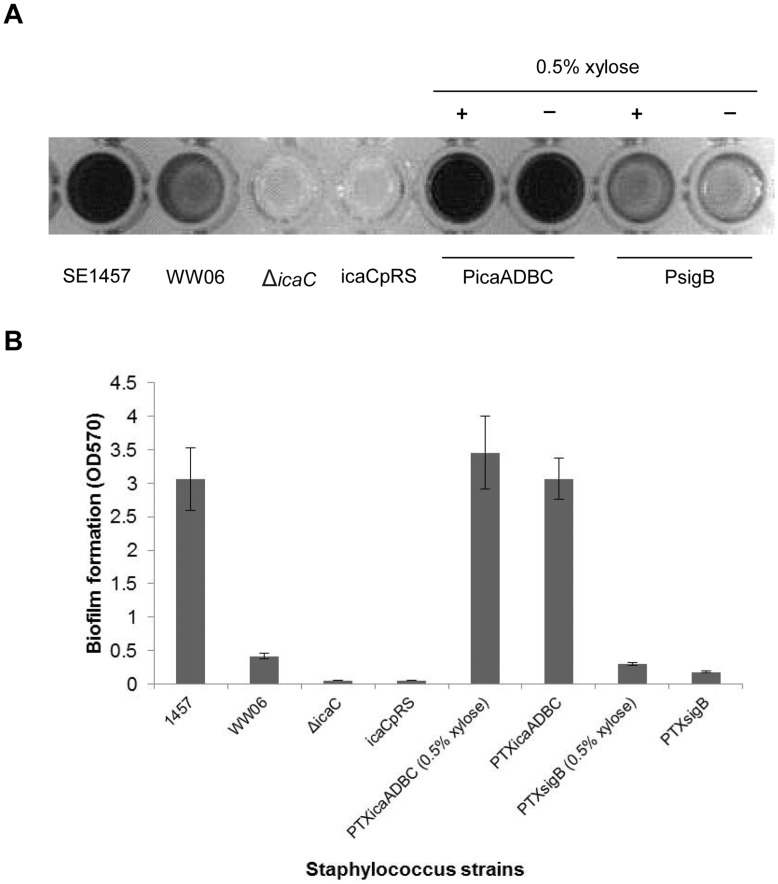
Biofilm formation in complementation strains PicaADBC, icaCpRS and PsigB in 96-well microtiter plates. (A) SE1457, WW06, Δ*icaC* and icaCpRS were cultivated in TSB; PicaADBC and PsigB were cultivated in TSB with or without 0.5% (g/ml) xylose. Biofilms formed over 24 h in 96-well microtiter plates were stained with 2% crystal violet. (B) The crystal violet was dissolved in ethanol and the absorbance was determined at 570 nm. The mean value ± standard deviation for each strain is shown.

### Detection of ArlR Expression in SE1457 and WW06 by Western Blot

Overnight cultures of *S. epidermidis* strains SE1457 and WW06 were inoculated in 100 ml TSB and incubated at 37°C for 4 h. After centrifugation, pellets were washed three times with distilled water and resuspended in 2 ml phosphate buffered saline. After incubation with 100 µg lysostaphin at 37°C for 1 h, cells were ultrasonicated and centrifuged. Supernatants were assayed for total protein concentration by Bradford method. One microgram of bacterial cell extracts were separated by sodium dodecyl sulfate polyacrylamide gel electrophoresis and then transferred to polyvinylidene fluoride membranes (GE-Whatman, Shanghai, China); purified rArlR (200 ng) was used as a control. Blots were probed with anti-rArlR antiserum (diluted 1∶1000) followed by horseradish peroxidase-labeled goat anti-mouse IgG (diluted 1∶2000) (Sangon, Shanghai, China). HRP activity was visualized via chemiluminescence detection using CSPD (Roche, Mannheim, Germany).

**Figure 7:**
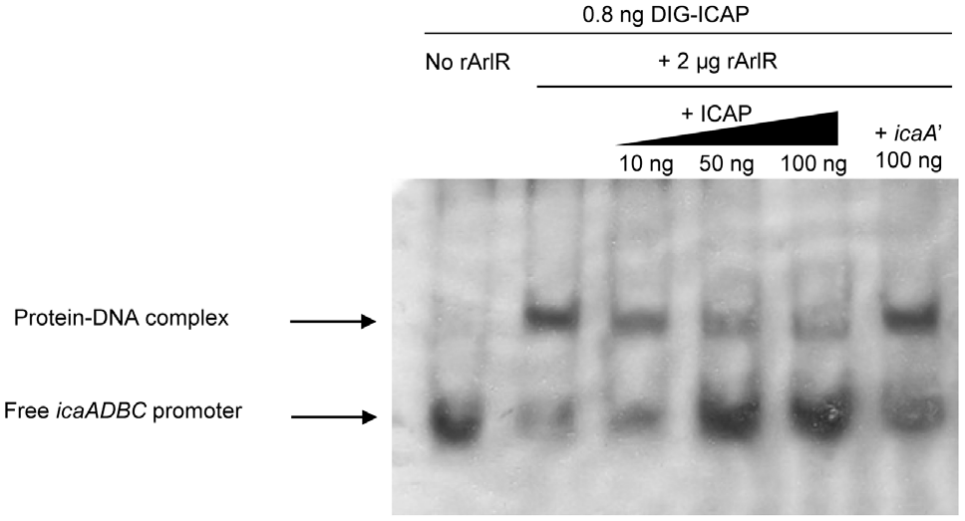
Binding of ArlR to the *icaADBC* promoter. Lanes were loaded as follows: lane 1, 0.8 ng Dig-ICAP alone; lane 2, 0.8 ng Dig-ICAP and 2 µg rArlR; lanes 3–5, Dig-ICAP, rArlR and increasing amounts of unlabeled ICAP (12.5, 62.5,125 fold increase in Dig-ICAP, respectively); and lane 6, Dig-ICAP, rArlR and 100 ng of a 405 bp *icaA* fragment. The DIG-labeled DNA fragments were transferred to positively charged nylon membranes and visualized by an enzyme immunoassay using anti-Digoxigenin-AP, Fab-fragments and the chemiluminescent substrate CSPD. Chemiluminescent signals were recorded on X-ray film. DIG-ICAP, digoxin-labeled *icaADBC* promoter region; ICAP, unlabeled *icaADBC* promoter region; icaA’, icaA gene fragment.

### Bacterial Initial Attachment Assay

The initial attachment ability of *S. epidermidis* cells was tested as described by Heilmann et al. [27].

### Semi-quantitative Detection of Biofilms

*S. epidermidis* biofilm formation was detected as previously described [28]. Overnight bacterial cultures grown in TSB were diluted 1∶200, then transferred to 96-well polystyrene microtiter plates (200 µl per well). After incubation at 37°C for 24 h, wells were washed gently three times with PBS and stained with 2% crystal violet for 5 min. Next, the plate was rinsed under running tap water, air-dried, and ethanol was used to dissolve the crystal violet; finally, absorbance was determined at 570 nm.

**Figure 8:**
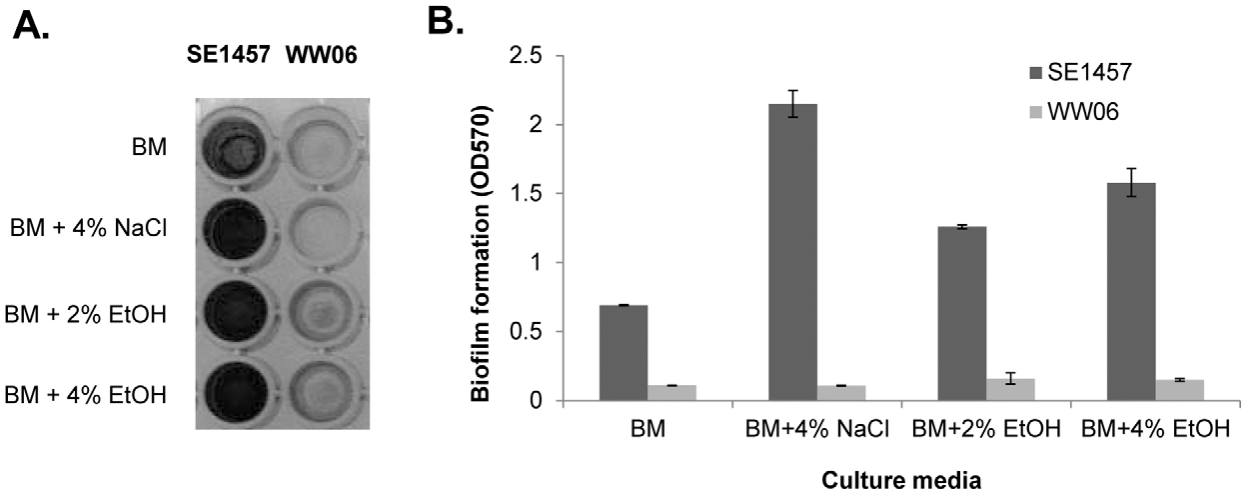
Effect of ethanol or sodium chloride on biofilm formation of SE1457 and WW06. (A) Semi-quantitative detection of biofilms formed by SE1457, WW06 in BM supplemented with 4% NaCl, 2% EtOH or 4% EtOH in 96-well polystyrene microtiter plates: The biofilms were stained with 2% crystal violet. (B) The crystal violet was dissolved in ethanol and the absorbance was determined at 570 nm. The mean value ± standard deviation for each strain is shown.

### Scanning Electron Microscope Observations of Biofilms

Overnight cultures of *S. epidermidis* strains were diluted in 1∶200 in TSB and added in 96-well plates containing fragments of a central vein catheter (ABLE, Guangdong, China). After incubation at 37°C for 24 h, catheter fragments were washed and fixed with 2.5% glutaric dialdehyde (pH 7.4), followed by a secondary fixation with 1% osmium tetroxide. Catheter fragments were then washed twice with distilled water, dehydrated in 70%, 95% and 100% ethanol, dried, fixed onto cylindrical metal plates and gilded with an ion sputter (HCP-2, Hitachi, Tokyo, Japan). Catheter fragments were observed for biofilm formation using a scanning electron microscope (XL-30, Philips, Eindhoven, Netherlands). SEM micrographs were taken at ×3000 and ×10000 magnifications.

### Biofilm Formation of the *arlRS* Mutant *in vivo*

Three New Zealand White female rabbits (2.0–2.5 kg) were anesthetized by intravascular injection with sodium pentobarbital (35 mg/kg of body weight). After the back of rabbit was denuded of fur, sterile polyethylene disks (1.0 cm diameter, 0.1 cm thickness, with 2 mm chimb) were implanted subcutaneously at four sites bilateral to the spine, as reported previously [29]–[31], three disks per site. About 10^8^ *S. epidermidis* cells from overnight cultures of SE1457, WW06 and ParlRS were collected, resuspended in TSB and injected subcutaneously into different implantation site respectively, using fresh TSB as a negative control. Three days after, the rabbits were euthanized and the implants were removed. The biofilms were scraped from the disks and the viable bacteria (colony forming units, CFUs) were determined. Differences in log_10_ CFU counts among groups were assessed with one-way analysis of variance (ANOVA) tests using software SPSS version 11.5 (SPSS Inc., Chicago, IL, USA).

### Semi-quantitative Detection of Polysaccharide Intercellular Adhesin (PIA)

PIA detection was performed as previously described [32] using wheat germ agglutinin (WGA) as a surrogate for anti-PIA antibody, using an *icaC* deletion mutant of SE1457 as a PIA negative control, and strain RP62A as a PIA positive control. Overnight cultures of SE1457, WW06, RP62A and Δ*icaC* in TSB supplemented with 0.5% glucose were diluted to an OD_578_ of 0.07 with fresh media, added into sterile 6-well cell cultures plates (6 ml/well) (Nunc, Roskilde, Denmark), and incubated at 37°C under static conditions. After 24 h incubation, cultures and the attached biofilms were transferred into 2 ml tubes and centrifuged. To prepare cell surface extracts for PIA detection, pellets were resuspended in 0.5 M ethylenediaminetetraacetic acid, pH 8.0 (3 ml per gram wet weight), followed by heating at 100°C for 5 min. After centrifugation, 40 µl of the supernatant was mixed with 10 µl of proteinase K (20 mg/ml) and incubated at 37°C for 3 h, followed by heating at 100°C for 10 min. Different dilutions of the extracts were transferred to a nitrocellulose membrane, using a 96-well dot blot system (Biometra, Goettingen, Germany). Membranes were blocked with 3% bovine serum albumin and incubated with 2 µg/ml HRP-labeled wheat germ agglutinin (WGA-HRP conjugate, Lectinotest Laboratory, Ukraine) for 1 h. HRP activity was visualized by chromogenic detection using 4-Chloro-1-Naphthol.

### Analysis of Transcriptional Levels of Biofilm-related Genes in WW06

To define the *arlRS* regulon, transcriptional levels of biofilm-related genes, *icaADBC, icaR, rsbU, sigB, luxS* and *sarA*, in the *arlRS*-deficient strain WW06 and the wild-type parent strain SE1457 were analyzed by RT-qPCR analysis using the ABI 7500 real-time PCR system. Gene-specific primers (Table 2) were designed to yield approximately 100 bp specific products; the housekeeping gene *gyrB* was used as an endogenous control. All samples were analyzed in triplicate and normalized against *gyrB* gene expression.

### Electrophoretic Mobility Shift Assay (EMSA)

Interaction of the recombinant ArlR and the *icaADBC* promoter was analyzed by EMSA using the DIG Gel Shift Kit (Roche). rArlR was phosphorylated prior to gel shift reaction by incubating rArlR with 50 mM acetylphosphate for 1 h. The DNA fragment upstream *icaADBC* (ICAP, 216 bp) was amplified and linked with digoxin-labeled dd-UTP. The resulting DNA fragment, Dig-ICAP, was used for EMSA in native PAGE. Lanes 1–2 were loaded with 0.8 ng Dig-ICAP alone and 0.8 ng Dig-ICAP mixed with 2 µg rArlR, respectively. In lanes 3–5, increasing amounts of unlabeled ICAP (12.5, 62.5, 125 fold increase in the amount of Dig-ICAP in lane 2, respectively) was added as a specific competitor to the mixture of Dig-ICAP and rArlR. In lane 6, 100 ng of a 405 bp *icaA* fragment was added as a nonspecific competitor. Primers used for to amplify partial *icaA* gene and ICAP are listed in Table 2. The DNA fragments were transferred to positively charged nylon membranes (Roche) by electro-blotting and visualized by an enzyme immunoassay following the manufacturer’s instruction.

### Cell Autolysis Assay

Autolysis assays for *S. epidermidis* strains were performed as previously described [33]. Strains SE1457, WW06, ParlRS and Δ*atlE* were cultured in TSB containing 1 M NaCl at 37°C to exponential phase (OD_580_ = 0.7). Additionally, WW06 and ParlRS were cultured in TSB containing 1 M NaCl and 2 µM CdCl_2_. Bacterial cells in 50 ml cultures were harvested by centrifugation, washed twice with ice-cold water and resuspended in 50 ml of 0.05 M Tris/HCl (pH 7.2) containing 0.05% (v/v) Triton X-100. Cells were then incubated at 30°C with shaking (200 rpm), and OD_580_ was measured at 30 min intervals.

## Results

### Construction of an *arlRS* Gene-deficient Mutant Strain of *S. epidermidis*

In *S. epidermidis* strain SE1457, the TCS *arlRS* consists of two genes, *arlS* and *arlR*, which share the same promoter and overlap by 4 bp. To investigate the function of *arlRS* in *S. epidermidis* biofilm formation, an ArlRS deficient mutant was constructed by replacing the *arlS* gene with an erythromycin-resistant cassette in the biofilm-forming SE1457 strain. The mutant was designated WW06. Western blot analysis of the whole cell extracts from WW06 using antiserum against the recombinant ArlR (rArlR, Figure S1) showed that no ArlR was expressed in WW06 (Figure S2). Thus, WW06 was an ArlRS-deficient mutant.

### ArlRS Deficiency in *S. epidermidis* Abolishing Biofilm Formation *in vitro*

The impact of ArlRS mutation on biofilm formation was investigated using both polystyrene microtiter plates and intravenous catheters. In the microtiter plates, biofilm formation in WW06 was dramatically decreased (OD_570_ = 0.41±0.04), compared to its wild-type counterpart (OD_570_ = 3.06±0.47) (Figure 1 A, B). To rule out the possibility of a polar effect, two complementation strains, ParlS and ParlRS, were constructed using plasmids expressing *arlS* gene and *arlRS* genes respectively. While ParlRS partially restored biofilm-forming ability (OD_570_ = 1.84±0.08), the biofilm-forming ability of ParlS (OD_570_ = 0.45±0.02) was similar to that of WW06.

To further investigate the ability of *S. epidermidis* strains WW06, ParlS, ParlRS, and SE1457 to form biofilms, these strains were incubated with catheter fragments in 96-well plates at 37°C for 24 h and biofilm formation morphology was assessed by SEM. The wild-type strain SE1457 generated a compact, thick biofilm on the catheter surface, whereas the mutant WW06 formed only a few bacterial cell clusters. ParlRS formed a flat biofilm on the catheter, whereas ParlS formed only a small number of microcolonies similar to WW06 (Figure 2).

In terms of bacterial growth, the growth curves of SE1457, WW06, ParlS and ParlRS were similar (Figure 1C). Additionally, in an assay measuring initial adherence, the numbers of attached cells of SE1457 and WW06 were 3.64×10^4^/cm^2^ and 3.58×10^4^/cm^2^, respectively, which showed that mutation of *arlRS* exhibited little effect on bacterial growth and primary attachment.

### Influence of ArlRS Deficiency on *S.epidermidis* Biofilm Formation *in vivo*

To investigate the effect of ArlRS deficiency on *S. epidermidis* biofilm formation *in vivo*, a biofilm infected rabbit model was established. The biofilm formation of SE1457, WW06 and ParlRS on the implanted disks *in vivo* was observed under an optical microscope and evaluated by determination of viable cells recovered from biofilms. SE1457 formed much thicker biofilms than WW06 on the implanted disks. The log_10_CFU/disk number of viable cells recovered from the biofilms of the *arlRS* mutant WW06 was significantly lower than that of SE1457 (3.08±0.42 vs 4.97±0.11, *P*<0.001) (Figure 3). The log_10_ number of viable CFU in biofilm of the complementation strain ParlRS was partially restored, which is higher than that of WW06 (4.02±0.25 vs 3.08±0.42, *P*<0.001).

### ArlRS Mutation Affecting Bacterial Autolysis

To investigate the effect of ArlRS on bacterial autolysis, SE1457, WW06 and ParlRS were incubated with 0.1% Triton X-100 for 2.5 h and the percent autolysis was calculated. The lytic percentage of WW06 reached 97.83%, which was significantly higher than the 55.38% lytic percentage for SE1457. The autolysis rate of ParlRS was similar to that of WW06 when cultured in media without cadmium chloride (CdCl_2_); however, when cultured with 2 µM CdCl_2_ to induce *arlRS* expression, the lytic percentage of ParlRS was similar to wild-type (Figure 4).

### Transcriptional Analysis of Biofilm-related Genes in WW06

To define the *arlRS* regulon and the role of ArlRS in *S. epidermidis* biofilm formation, total RNAs from the *arlRS*-deficient strain WW06 and the wild-type parent strain SE1457 in mid-log growth phase (4 h) were extracted, and transcriptional levels of several biofilm-related genes, *rsbU, sigB, sarA, icaR, luxS* and *icaA,* were analyzed by RT-qPCR, using the housekeeping gene *gyrB* as an internal control. The time point was selected because *arlRS* transcription in SE1457 reached peak levels at 4 h [34].

Transcription of the biofilm-related genes *rsbU, sigB, sarA* and *icaA* in WW06 were found to be down-regulated 14.5 fold, 8.4 fold, 4.3 fold and 7.5 fold, as compared to SE1457, whereas *icaR* transcription was upregulated 2.4 fold and expression of the quorum sensing-related gene *luxS* was similar (Figure 5A).

### ArlRS Involving in *S. epidermidis* Biofilm Formation in an *ica*-dependent Manner

In contrast to *S. aureus*, where the ArlRS TCSs has been implicated in *ica*-independent biofilm formation, RT-qPCR analysis of the ArlRS-deficient mutant of *S. epidermidis* showed down-regulation of *icaADBC* transcription. To determine whether the down-regulation of *icaADBC* expression in WW06 functionally impacted biofilm formation, PIA formation was examined. SE1457 and WW06 were grown under conditions favoring biofilm formation and PIA concentrations were analyzed by a semi-quantitative dot blot assay; using a non-biofilm forming strain Δ*icaC* as a negative control and strong biofilm producing strain RP62A as a positive control. As expected, no surface-located PIA was detected from either WW06 or Δ*icaC*, whereas large amount of PIA production was observed in both SE1457 and RP62A. PIA synthesis was restored in the complementation strain ParlRS (Figure 5B).

To confirm that ArlRS is an upstream regulator of *ica*-dependent biofilm formation in *S. epidermidis*, the *arlRS* mutant was complemented with *icaADBC* on a plasmid under the control of xylose inducible promoter (pTX-*icaADBC*). This complementation restored biofilm-forming ability. In the contrast, overexpressing *arlRS* in the Δ*icaC* mutant did not recover biofilm formation (Figure 6). Although transcription of the *sigB* gene was down-regulated in WW06, when the mutant was complemented with a plasmid-encoding *sigB* gene (pTX-*sigB*), no obvious differences in biofilm formation were observed (Figure 6).

As ArlR is a DNA binding protein, we hypothesized that ArlR may bind directly to the promoter region of *icaADBC* to modulate transcription of the operon. Thus, EMSA were performed with digoxin-labeled DNA directly upstream of *icaADBC* (ICAP, 216 bp) and recombinant ArlR (rArlR). As shown in Figure 7, rArlR bound to Dig-ICAP and formed a DNA-protein complex, shifting Dig-ICAP behind (lane 2) compared to Dig-ICAP alone (lane 1). When increasing amounts of unlabeled ICAP was added as a specific competitor, more free Dig-ICAP was observed (lanes 3–5), while when a nonspecific competitor, *icaA* fragments, were added, no Dig-ICAP was shifted back (lane 6). It indicated a potential mechanism for ArlRS regulation of *ica* expression.

To further investigate the role of IcaR in regulating WW06 biofilm formation, effect of ethanol or sodium chloride on biofilm production of WW06 and SE1457 was detected. Addition of 4% NaCl to BM media increased SE1457 biofilm formation (approximately 3.1 fold), while had no effect on that of WW06, shown in Figure 8. SE1457 showed enhanced biofilm production in BM media supplemented with either 2% or 4% ethanol (1.8 fold or 2.3 fold, respectively), whereas ethanol showed very limited effect on WW06 biofilm formation (1.3 fold −1.4 fold increase).

## Discussion

The two-component signal transductionsystem ArlRS is a global regulator of virulence genes in *S. aureus* [19], modulating the extracellular proteolytic activity, capsule formation, biofilm formation and production of Protein A, α-toxin, β-hemolysin, lipase, coagulase and serine protease. However, the function of ArlRS in *S. epidermidis* was unclear. Thus, we investigated the role of ArlRS in *S. epidermidis*, an opportunistic pathogen that lacks many virulence genes [35] but can form biofilms as a major pathogenic factor. First, an ArlRS deficient mutant WW06 was constructed. WW06 showed dramatically decreased biofilms formation *in vitro* and *in vivo*. No obvious difference was found in either growth curves or initial attachment of WW06 and SE1457. Extracellular DNA has been reported to play an important role in biofilm formation [36]–[38]. In this study, however, the quantity of eDNA in the unwashed biofilms of the two strains was similar (data not shown), although the *arlRS* mutant strain WW06 exhibited a significantly higher autolysis rate than SE1457. Thus, we hypothesize that ArlRS modulates *S. epidermidis* biofilm formation by regulating the expression of the biofilm-related genes involved in the step of intercellular adhesion.

Many factors are involved in the second step of biofilm formation, e.g. polysaccharide intercellular adhesin (PIA) [39], accumulation associated protein (Aap) [40], [41], Extracellular matrix binding protein (Embp) [42], etc. The *ica* (intercellular adhesin) operon plays a crucial role by synthesizing PIA that is a main component of the extracellular polymeric substances (EPS). The *ica* operon is composed of the *icaR* (regulatory) gene and *icaADBC* (biosynthesis) genes. The *icaR* gene is located upstream of the *icaADBC* and is transcribed divergently, encoding a transcriptional repressor that negatively regulates *icaADBC* transcription in both *S. aureus* and *S. epidermidis.* Staphylococcal biofilms formation involves *ica*-dependent and *ica*-independent pathways [43]. In the present study, we have demonstrated that ArlRS activates *icaADBC* transcription, leading to PIA production and *S. epidermidis* biofilm formation. First, RT-qPCR analysis showed that *icaADBC* expression was significantly reduced in the ArlRS*-*deficient mutant WW06. Secondly, whereas semi-quantitative PIA detection revealed strong PIA production in the wild-type strain SE1457, little or no PIA expression was detected in WW06 mutant. Furthermore, complementation of *icaADBC* in WW06 restored its ability to form biofilms, whereas overexpression of *arlRS* in the *icaC* deletion mutant of SE1457 (Δ*icaC*) did not recover biofilm formation. Together, these results indicate that *icaADBC* is the downstream biofilm-related effector in the ArlRS regulation pathway. Moreover, EMSA demonstrated that recombinant ArlR binds to the 216-bp region located upstream of *icaADBC*. Overall, it reveals that ArlRS regulates *S. epidermidis* biofilm formation in an *ica*-dependent manner.

Previous studies have showed that ArlRS promotes biofilm formation in *S. aureus* strain ISP794 and strain 15981 which is capable of producing strong biofilms in TSB while unable to produce a biofilm in HHWm [18], [22]. Fournier, B. et, al. have found that an *arlS* gene transposition mutant (BF16) of *S. aureus* clinical strain ISP794 exhibited an increased ability to form biofilms on polymer surfaces, resulting from enhanced bacterial autolysis and altered peptidoglycan hydrolase activity. In the present study, *arlRS* mutation in *S. epidermidis* resulted in increased Triton X-100 induced bacterial autolysis, but decreased biofilm formation. The difference of altered biofilm formation between *arlS* mutant of *S. aureus* (BF16) and *arlRS* mutant of *S. epidermidis* (WW06) could be explained that due to different methods used for gene mutation (transposon insertion in the former and gene knockout in the latter), ArlR is absent in WW06 while present and may have function in BF16. Toledo-Arana, A. et, al. have reported that deletion of the *arlRS* locus in *S. aureus* clinical strain 15981 enhanced initial cell attachment and provoked accumulation of PNAG and biofilm formation when cultured in Hussain-Hastings-White-modified medium (HHWm). However, in HHWm the biofilm formation of the *arlRS* and *icaADBC* operon double mutant increased at the same level as the *arlRS* mutant, suggesting ArlRS is involved in early stage of strain 15981’s biofilm development in HHWm in an *ica*-independent manner. Interestingly, mutation of *arlRS* in *S. epidermidis* resulted in decreased biofilm formation in TSB in an *ica*-dependent manner. Bioinformatics analysis showed that *arlR* and *arlS* genes are conserved in *S. epidermidis* (over 95% nucleotide sequence identities among strains RP62A, SE1457 and ATCC12228) while *S. epidermidis* strain RP62A and *S. aureus* strain COL [44] share relatively high nucleotide sequence identities of *arlR* and *arlS* genes (74.8% and 70%, respectively). It appears ArlRS is involved in different mechanisms that regulate biofilm formation in the closely related species.

Current findings indicate that *icaADBC* expression in *S. epidermidis* is regulated by multiple regulatory factors including RsbU, SigB, IcaR, SarA and LuxS[8], [45]–[48]. Knobloch, J.K. et, al. reported that inactivation of *rsbU* gene (a positive regulator of *sigB*) in *S. epidermidis* was associated with both a reduction in PIA levels and biofilm formation, and the regulation of biofilm formation by *rsbU* is SigB dependent. Handke, L.D. et, al. reported that *icaADBC* transcription is down-regulated in the *sigB* mutant [45]. The regulation of *icaADBC* transcription by SigB was found to be mediated through the upregulation of IcaR expression. IcaR is a transcriptional repressor of *icaADBC* [49]. The *icaR* gene deletion in *S. epidermidis* strain O-47 [50] led to increased PIA synthesis. In the present study, decreased transcription of *rsbU*, *sigB* and *icaADBC,* and increased *icaR* expression are observed in the *arlRS* mutant WW06, compared with SE1457. The results are consistent with the previous findings, indicating a potential mechanism that the regulation of *icaADBC* expression by ArlRS may be mediated by IcaR. The global transcriptional regulator SarA plays an important role in staphylococcal biofilm formation [8], [51]–[54]. Transcription of *sarA* was decreased by about 4.3 fold in the WW06 mutant, which is consistent with previous findings that SarA positively regulates *ica* operon expression in an *icaR*-independent manner; its role in ArlRS regulation pathway requires further investigation. As a quorum sensing system, LuxS repressed *S. epidermidis* biofilm formation through a cell-cell signaling mechanism based on autoinducer 2 secretions. LuxS affects biofilm formation by altering production of PIA via transcriptional regulation of the *ica* operon. In the present study, transcriptional levels of *luxS* in the SE1457 and WW06 were similar, indicating *luxS* may not play a role in the ArlRS regulation pathway in *S. epidermidis*.

Taken together, it reveals that in *S. epidermidis*, the ArlRS two-component signal transduction system is involved in the modulation of bacterial autolysis and in the regulation of biofilm formation an *ica*-dependent manner, which is distinct from the role of ArlRS in *S. aureus* and deserves further study.

## Supporting Information

Figure S1 **Purification of the recombinant ArlR by affinity chromatography.** The *arlR* gene was cloned in the expression vector pET28a(+) to form pET-*arlR*, which was transformed in the *E. coli* BL21(DE3+). After induction with 0.4 mM IPTG for 12 h, the recombinant ArlR was purified by affinity chromatography. M: Protein molecular weight marker; rArlR: the purified recombinant ArlR (about 27 kDa).
(TIF)

Figure S2 **Detection of ArlR expression in SE1457 and WW06 by Western blot.** The lanes were loaded with 200 ng purified recombinant ArlR (rArlR), 1 µg bacterial cells extract of WW06, and 1 µg bacterial cells extract of SE1457, respectively. Antiserum from the mouse immunized with 5 µg recombinant ArlR was diluted by 1∶1000. An unspecific protein band with a lower molecular mass was present in each lane.
(TIF)
