# Supplementary figures and images for: The Two-Component Signal Transduction System ArlRS Regulates Staphylococcus epidermidis Biofilm Formation in an ica-Dependent Manner
Source: PLoS One. 2012 Jul 27;7(7):e40041. doi: 10.1371/journal.pone.0040041 (PMC3407220; doi:10.1371/journal.pone.0040041)

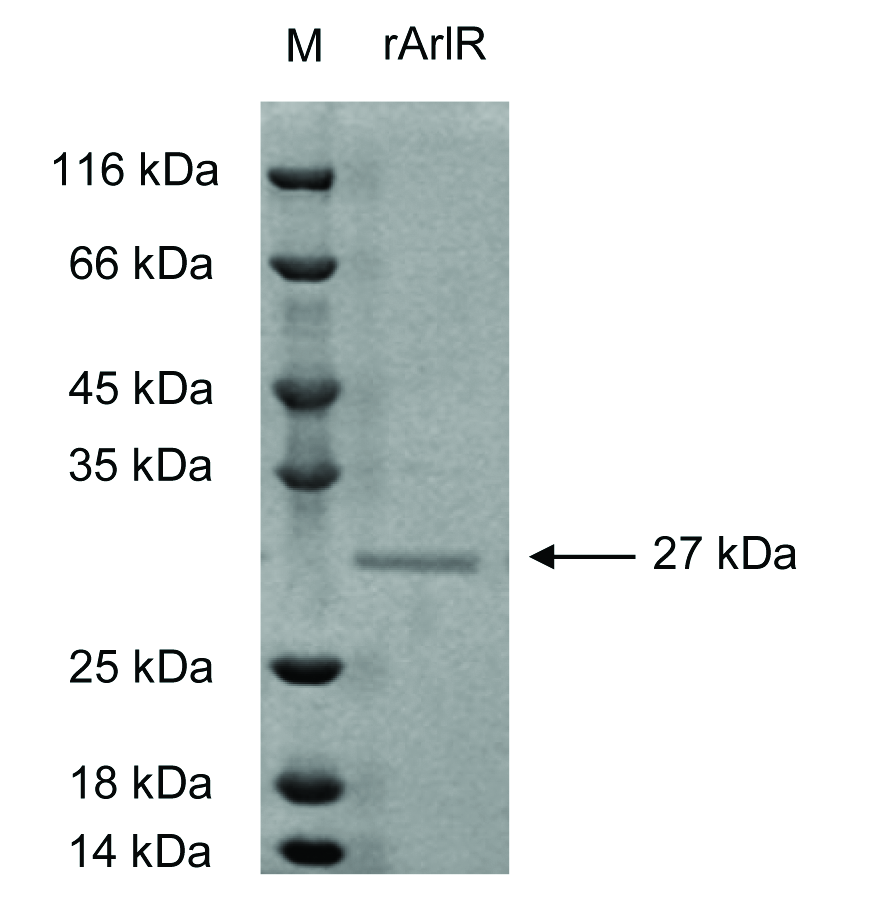

Supplement: Figure S1 — Purification of the recombinant ArlR by affinity chromatography. The arlR gene was cloned in the expression vector pET28a(+) to form pET-arlR, which was transformed in the E. coli BL21(DE3+). After induction with 0.4 mM IPTG for 12 h, the recombinant ArlR was purified by affinity chromatography. M: Protein molecular weight marker; rArlR: the purified recombinant ArlR (about 27 kDa). (TIF) [file pone.0040041.s001.tif]

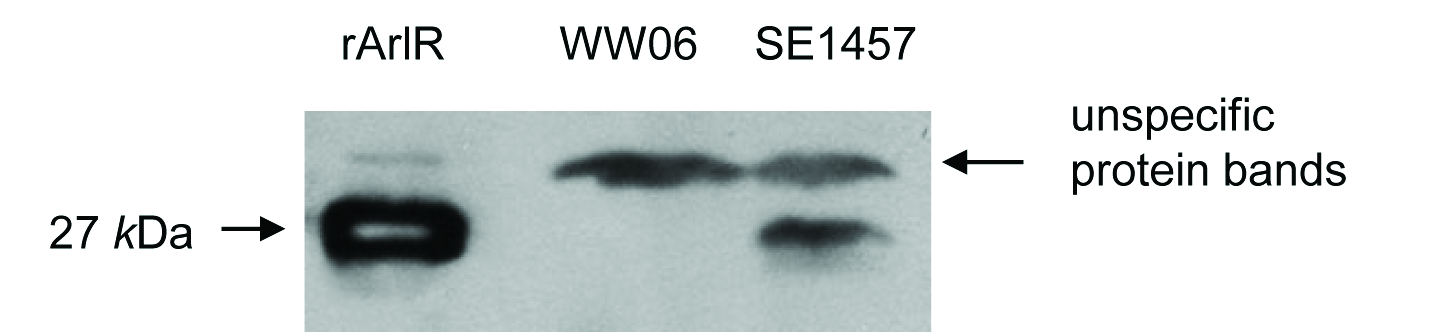

Supplement: Figure S2 — Detection of ArlR expression in SE1457 and WW06 by Western blot. The lanes were loaded with 200 ng purified recombinant ArlR (rArlR), 1 µg bacterial cells extract of WW06, and 1 µg bacterial cells extract of SE1457, respectively. Antiserum from the mouse immunized with 5 µg recombinant ArlR was diluted by 1∶1000. An unspecific protein band with a lower molecular mass was present in each lane. (TIF) [file pone.0040041.s002.tif]
